# Let-7c-5p Is Involved in Chronic Kidney Disease by Targeting TGF-*β* Signaling

**DOI:** 10.1155/2020/6960941

**Published:** 2020-06-12

**Authors:** Zhenguo Wang, Cuixing Zhou, Yangyang Sun, Yimeng Chen, Dong Xue

**Affiliations:** Department of Urology, The Third Affiliated Hospital of Soochow University, Changzhou 213003, China

## Abstract

The purpose of the present study was to investigate the expressions of hsa-let-7c-5p and TGF-*β* signaling-related molecules and their correlations with clinical characteristics in chronic kidney disease (CKD). Twenty-three biopsy specimens of CKD patients and 20 negative control tissues were selected. Quantitative real-time PCR (qPCR) was used for the detection of hsa-let-7c-5p, transforming growth factor *β* (TGF-*β*) and TGF-*β* receptor type 1 (TGF-*β*R1) expression levels. Target gene of hsa-let-7c-5p was verified by dual-luciferase reporter assay. A significant decrease of hsa-let-7c-5p expression in CKD tissue was found, compared with that of normal renal tissues (*p* < 0.01). Expression levels of TGF-*β* in CKD were increased, compared with that of normal kidney tissue (*p* < 0.001). The difference in the expression of TGF-*β* R1 between CKD tissues and normal renal tissues was not significant (*p* > 0.05). A negative correlation was found between the expression of TGF-*β* and renal tissue hsa-let-7c-5p levels. Furthermore, hsa-let-7c-5p was identified to regulate TGF- *β*1 by directly binding with the 167-173 site in the 3′ untranslated region. Decreased hsa-let-7c-5p levels in CKD patients was found to be associated with disease severity, which shows a negative correlation with proteinuria and creatinine levels, and a positive correlation with estimated glomerular filtration rate (eGFR), while relative TGF-*β*1 expression had a positive correlation with creatinine level. In summary, changes in hsa-let-7c-5p expression and its target gene TGF-*β* are associated with the disease status of CKD. Let-7c-5p may contribute to the pathogenesis of renal fibrosis through TGF-*β* signaling, a potential diagnostic and therapeutic target of the disease.

## 1. Background

During the past 10 years, chronic kidney disease (CKD) has been highlighted as a leading public health problem. In 2010, the overall prevalence of CKD in China was 10.8%, and China is estimated to have around 119.5 million CKD patients [1]. The latest research indicates that the overall prevalence of CKD in Chinese rural residents was 16.4% [2]. The progression of renal pathological changes in CKD eventually leads to end-stage renal failure [3, 4]. End-stage renal disease patients worldwide are increasing, at a rate of 7-8% each year [5]. CKD has become a prominent public health problem after cardiovascular and cerebrovascular diseases, cancer, and diabetes in China.

TGF-*β* is a multifunctional polypeptide cytokine, which is widely expressed in various tissues, such as connective tissue, liver, kidney, lung, brain, skin, and muscles [6]. TGF-*β* signaling disruption is associated with various developmental disorders and numerous human diseases, including autoimmune diseases, fibrosis, and cancer. As a key profibrotic cytokine, TGF-*β* plays a primary role in the induction and progression of CKD. TGF-*β*1 can induce renal fibrosis via activation of both canonical (Smad-based) and noncanonical (non-Smad-based) signaling pathways [7]. The TGF-*β*/Smad signaling pathway is the most important tissue fibrosis regulatory pathway [7–9]. Intracellular signal transduction mediated by TGF-*β* and Smad proteins can not only promote the deposition of extracellular matrix (ECM) but can also inhibit its degradation. Therefore, it may destroy the dynamic balance of ECM aggregation and degradation and promote the occurrence of fibrosis, which is closely related with the sclerosis of various organs [6, 10–12].

With the deeper understanding of the pathogenesis of CKD, the identification of early-stage sensitive diagnostic markers has received increasing attention. MicroRNAs (miRNAs) are a class of ~22-nt noncoding RNAs that normally silence gene expression via translational repression and/or degradation of targeted mRNAs in eukaryotes [13]. Let-7 is one of the most widely studied miRNAs. It was first found in C.elegans as an important developmental regulator [14]. Many studies have demonstrated the implication of the let-7 family in the occurrence and progression of CKD [15–17]. Wang et al. reveal that let-7b exerts an antifibrotic response by downregulating TGFR1 expression and thereby limiting TGF-*β*1-induced canonical and non-canonical signaling [18]. Another study reports lipoxins attenuate renal fibrosis in rats with unilateral ureteric obstruction (UUO) model by inducing let-7c [15]. As a member of the let-7 family, hsa-let-7c-5p is important for cell growth and proliferation [19]. Although hsa-let-7c-5p is associated with various pathological processes, its renal fibrosis regulatory role has been rarely reported.

## 2. Material and Methods

### 2.1. Patients and Samples

Twenty-three patients with CKD diagnosed at our hospital from March to September 2018 were enrolled in this study. Irrespective of the underlying cause, individuals with an estimated glomerular filtration rate (eGFR) of <60 mL/min per 1.73 m^2^, or markers of kidney damage, or both, for at least 3 months in duration, were regarded as suffering from CKD [4]. Then, kidney biopsy was conducted, and pathological results confirmed that all patients were suffering from CKD. The pathological results of 23 patients were IgA nephropathy (*n* = 8), membranous nephropathy (*n* = 6), nephrotic syndrome (*n* = 4), amyloidosis nephropathy (*n* = 1), obesity-related glomerulopathy (*n* = 1), lupus nephritis (*n* = 1), diabetic nephropathy (*n* = 1), and proliferative sclerosing glomerulonephritis (*n* = 1).

Twenty kidney tissue specimens were collected during radical nephrectomy to be used as the negative control group. All kidney tissues in the control group were taken from areas far away from the kidney tumor and immediately stored in nitrogen canister. The institutional review board of our hospital gave approval to conduct the study, and all participants provided informed written consent. Creatinine, cystatin C, carbamide, proteinuria, hemoglobin, and albumin levels in serum were measured, and standard laboratory methods (Instrumentation Laboratory, Changzhou, China) were used to analyze the urine samples. Sysmex UC3500 urine dry chemistry analyzer and MEDITAPE UC-11A test paper were used to measure urine protein. The results were reported as semiquantitative results, with grades of 15 (±), 30 (1+), 100 (2+), 300 (3+), and 1000 (4+), in mg/dL.

Calculation of the eGFR was done using the CKD-EPI equation [20], based on serum creatinine level, sex, and age. eGFR (mL/min per 1.73 m^2^) = 141 × min (Scr/*κ*, 1) *α* × max (Scr/*κ*, 1) − 1.209 × 0.993 Age × 1.018 [if female]_1.159 [if black], where *α* is -0.329 for females and -0.411 for males, *κ* is 0.7 for females and 0.9 for males, Scr is serum creatinine level, while min and max indicate the minimum and maximum, respectively, of Scr/*κ*or 1. Demographics and clinical characteristics of participants are summarized in Table 1.

### 2.2. RNA Extraction and RT-qPCR

TRIzol reagent was used to extract total RNA from CKD tissue and normal kidney tissue. A NanoDrop 1000 spectrophotometer (Thermo Fisher Scientific) was employed for RNA concentration and purity determination. The purity of the RNA met the experimental requirement when the OD260/280 value was around 1.8.

A PrimeScript RT reagent Kit with gDNA Eraser (Takara, Japan) was used to perform the reverse transcription. Universal reverse transcription primers and miRNA-specific reverse transcription primers were used for the reverse transcription of mRNA and miRNA, respectively.

Real-time fluorescence quantitative PCR: detection and quantification of miRNA, mRNA, and internal reference genes were carried out using SYBR Premix Ex Taq (TliRNaseH Plus) (Takara, Japan). cDNA was amplified on a 7500 fluorescent quantitative PCR machine (Applied Biosystems, USA). The number of copies of *β*-actin and RNU6B was used as the correction base for mRNA and hsa-let-7c-5p expression, respectively. The comparative Ct (*ΔΔ*Ct) method was utilized for gene expression level calculation of each experimental group [22]. Therefore, the gene expression ratio in the experimental group relative to the control group was 2^-*ΔΔ*Ct^ after being standardized with the internal control gene.

### 2.3. miRNA Mimics and Cell Transfection

Ribobio Company (Guangzhou, China) was the source of the miRNA mimics purchased. The sequences of C. elegans, confirmed to show minimal homology to all known miRNAs of miRBase 18.0, were used as the base for the universal negative control for mimics. The HK-2 cells, a proximal tubular cell line derived from normal kidney, were purchased from American Type Culture Collection. HK-2 cells were maintained in 10% fetal bovine serum and 100 U/mL penicillin/streptomycin supplemented RPMI Medium 1640. A lipofectamine 3000 (Invitrogen) system was used as recommended by the manufacturer to transfect the cells. The final transfection concentration was 50 nM, and the subsequent treatment was conducted 48 hours after transfection. The experiment was repeated three times independently.

### 2.4. Statistical Analysis

Statistical analyses were done using SPSS 13.0 software, with the homogeneity of the normal variance of the data being analyzed. The results are expressed as mean ± standard deviation. The results of the two groups were compared using a *t*-test, and correlation analysis was carried out using the Pearson correlation analysis. The results of three or more groups were compared using one-way analysis of variance (ANOVA). *p* value <0.05 shows that the difference is statistically significant.

## 3. Results

### 3.1. The Relative Expressions of Hsa-Let-7c-5p, TGF-*β*1, and TGF-*β*R1 in CKD Tissues

**Figure 1:**
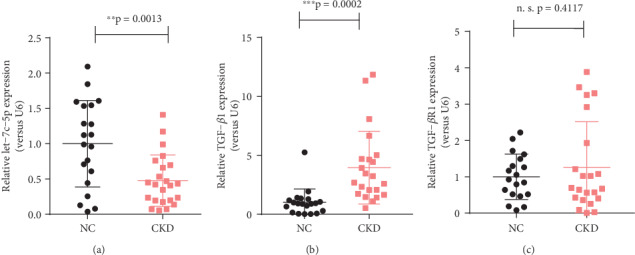
Dot-plots present the distribution of normalized expressions of (a) hsa-let-7c-5p, (b) TGF-*β*1, and (c) TGF-*β*R1 between CKD tissues (*n* = 23) and healthy control samples (*n* = 20). ∗∗*p* < 0.01; ∗∗∗*p* < 0.001; n.s.: not significant.

A significant decrease of hsa-let-7c-5p expression was found in CKD tissue compared with that of normal renal tissues, along with a marked statistically difference (*p* < 0.01, Figure 1(a)). TGF-*β*1 expression in CKD tissues was higher than that of normal kidney tissue, with a highly significant difference (*p* < 0.001, Figure 1(b)). However, no significant difference in CKD tissue TGF-*β*R1 expression, compared with normal renal tissues was detected (*p* > 0.05, Figure 1(c)). The relative expressions of hsa-let-7c-5p, TGF-*β*1, and TGF-*β*R1 are listed in Table 2.

### 3.2. Correlation Analysis of Hsa-Let-7c-5p and TGF-*β* Signaling and Clinical Applications in CKD Diagnosis

**Figure 2:**
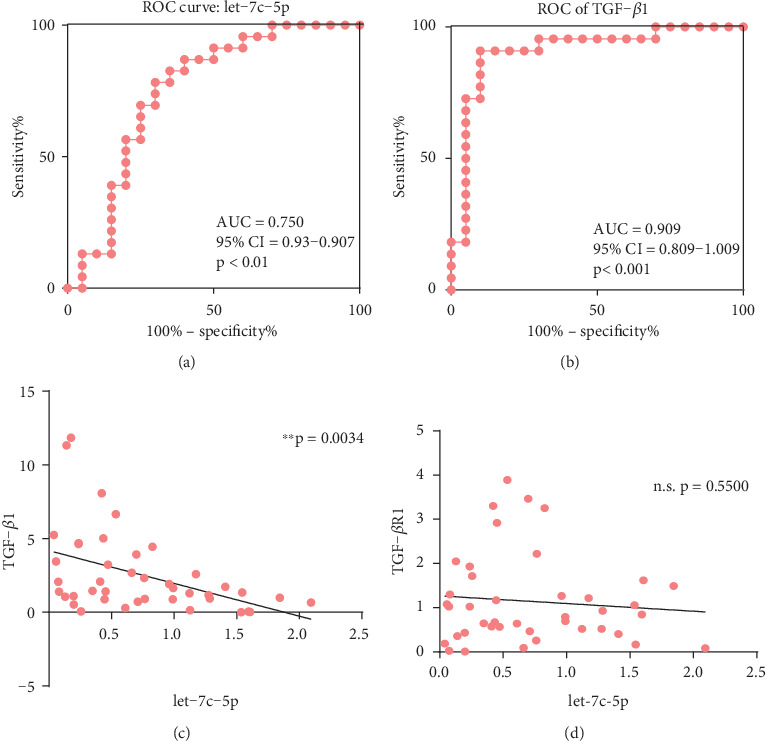
Correlation analysis of hsa-let-7c-5p and TGF-*β* signaling and their diagnostic potential. (a, b) Area under the ROC curve for hsa-let-7c-5p and TGF-*β*1 levels, respectively, between 23 CKD patients and 20 normal controls. (c) hsa-let-7c-5p expression is remarkably negatively correlated with TGF-*β* expression (*p* < 0.01, Pearson *r* = −0.4417). (d) hsa-let-7c-5p expression was not significantly correlated with TGF-*β* R1 levels. ∗∗*p* < 0.01; n.s.: not significant.

In order to further discover the potential clinical applications, abnormally expressed hsa-let-7c-5p and TGF-*β*1 levels between 23 CKD patients and 20 normal controls are illustrated on a receiver operating characteristic (ROC) curve. hsa-let-7c-5p was found to be sensitive and specific enough to identify CKD patients from among healthy controls, as shown by the ROC curve (sensitivity = 78.26%, specificity = 70.00%, Figure 2(a)). Moreover, the ROC curve of TGF-*β*1 shows a more pronounced distinction between normal and diseased subjects, with a value of 0.909 being the area under the curve (AUC) (sensitivity = 90.91%, specificity = 90.00%, Figure 2(b)).

Meanwhile, we found that hsa-let-7c-5p is remarkably negatively correlated with the expression of TGF-*β* (∗∗*p* < 0.01, Pearson *r* = −0.4417, Figure 2(c)) and shows no significant correlation with TGF-*β*R1 expression (*p* > 0.05, Figure 2(d)).

### 3.3. TGF-*β*1 Is a Potential Target Gene for Hsa-Let-7c-5p

**Figure 3:**
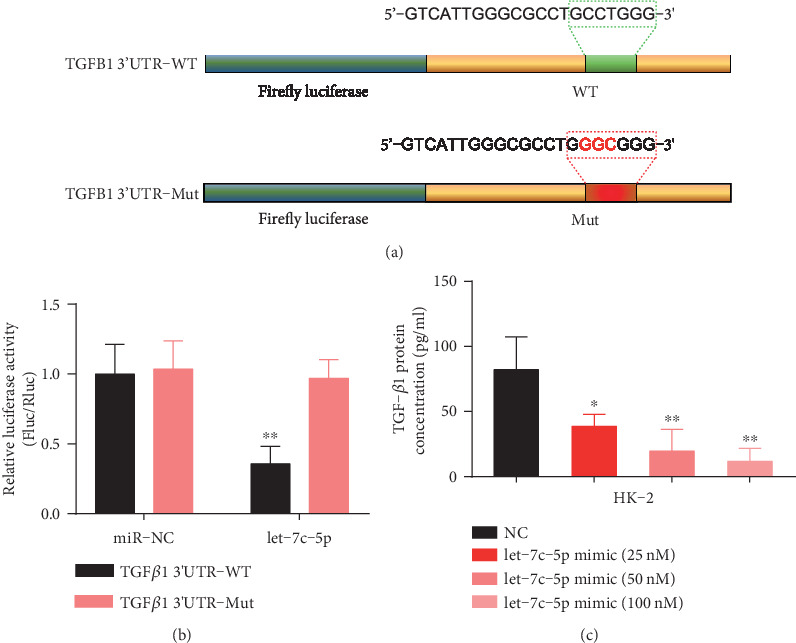
Identification of hsa-let-7c-5p as a regulator of TGF-*β*1 expression. (a) Sequence of wild-type (WT) and mutated (mutant) plasmids for dual-luciferase assay. (b) Relative luciferase activity of WT and mutated plasmids when transfected with miR-NC or let-7c-5p mimics. The ratio of renilla luciferase activity to firefly luciferase activity is shown. (c) TGF-*β*1 protein expression concentration at different dosages of hsa-let-7c-5p mimics, as evaluated using ELISA. ∗*p* < 0.05; ∗∗*p* < 0.01. Bar results are expressed as mean ± standard deviation.

Since TGF-*β*1 expression is negatively correlated with that of hsa-let-7c-5p (Figure 2(c)), we speculated the direct posttranscriptional regulation of TGF-*β*1 expression by hsa-let-7c-5p. There are several binding sites of hsa-let-7c-5p in the 3′ untranslated region (3′ UTR) of human TGF-*β* mRNA that were identified through bioinformatics analyses. In order to confirm the direct binding of hsa-let-7c-5p to TGF-*β*1 3′UTR, the predicted binding sites were mutagenized as shown in Figure 3(a). TGFB1 3′UTR-WT and TGFB1 3′UTR-Mut plasmids were generated by inserting a wild type or mutagenized TGF-*β*1 3′UTR sequence after the firefly luciferase sequence. Dual-luciferase assays were used to calculate the renilla luciferase activity to firefly luciferase activity ratio. hsa-let-7c-5p was not able to repress luciferase activity when mutagenized TGF-*β*1 3′UTR 167-173 position was applied (Figure 3(b)), indicating that the predicted sequence is indeed the genuine hsa-let-7c-5p binding site.

Furthermore, hsa-let-7c-5p was overexpressed by the transient transfection of hsa-let-7c-5p mimics, and the level of TGF-*β*1 protein was analyzed using ELISA. HK-2 cells, which express significant amounts of endogenous TGF-*β*1, were used in experiments. Overexpression of hsa-let-7c-5p resulted in a dose-dependent decrease in TGF-*β*1 protein levels (Figure 3(c)). These results further confirm that hsa-let-7c-5p regulates human TGF-*β*1 expression.

### 3.4. Correlation between the Expression of Hsa-Let-7c-5p and Clinicopathological Factors in Patients with CKD

**Figure 4:**
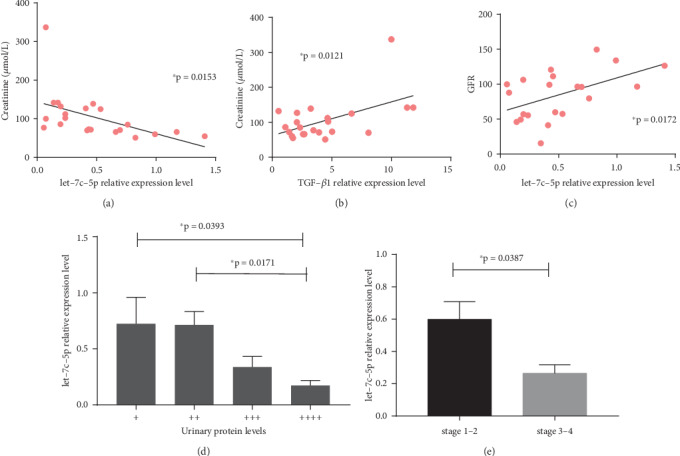
Correlation between hsa-let-7c-5p expression and clinicopathological factors of patients with CKD. (a) Serum creatinine levels are negatively correlated with relative hsa-let-7c-5p expression levels. (b) Serum creatinine levels are positively correlated with relative TGF-*β*1 expression. (c) hsa-let-7c-5p expression levels show a positive correlation with CKD patient eGFR. (d) Patients with proteinuria (4+) have significantly decreased hsa-let-7c-5p levels, in comparison with patients with proteinuria (+) and proteinuria (2+). (e) Significant decrease in hsa-let-7c-5p levels of stage 3-4 CKD patients, compared with that of early-stage patients. ∗*p* < 0.05. Bar results are expressed as mean ± standard deviation.

Serum creatinine level was found to be negatively related with the relative hsa-let-7c-5p expression level (*p* < 0.05, Pearson *r* = −0.5102, Figure 4(a)). Meanwhile, serum creatinine level was found to be positively related with the relative expression of TGF-*β*1 (*p* < 0.05, Pearson *r* = 0.5250, Figure 4(b)).

In order to investigate the correlation between the expression of hsa-let-7c-5p and disease severity in CKD patients, hsa-let-7c-5p expression level was analyzed against eGFR, proteinuria level, and stage of CKD. This shows that the expression level of hsa-let-7c-5p is positively related to the eGFR of CKD patients (*p* < 0.05, Pearson *r* = 0.5140, Figure 4(c)). CKD group patients were then divided into four subgroups based on their levels of proteinuria, and hsa-let-7c-5p expression among the different groups were compared. The expression of hsa-let-7c-5p continuously decreased as proteinuria levels increased. Patients with proteinuria (4+) were found to have significantly decreased hsa-let-7c-5p levels, in comparison with that of proteinuria (+) and proteinuria (2+) patients (Figure 4(d)). In addition, CKD patients were divided into subgroups based on their eGFR levels. Individuals with an eGFR of >60 mL/min per 1.73 m^2^ and/or markers of kidney damage, present for a minimum of 3 months in duration were regarded as stage 1-2 CKD patients, whereas those with an eGFR of <60 mL/min per 1.73 m^2^, lasting for at least a 3 months duration were regarded as stage 3-4 CKD patients [4]. hsa-let-7c-5p levels were found to have a significantly decrease in stage 3-4 CKD patients, compared with that of early stage patients (Figure 4(e)), indicating that hsa-let-7c-5p levels are related with disease severity.

## 4. Discussion

At the histological level, the progression of CKD results in tubulointerstitial and glomerular fibrosis owing to excessive deposition of ECM, leading to end-stage renal disease, irrespective of the initial injury [23, 24]. As mentioned above, TGF-*β*1 is a major regulator of kidney fibrosis. However, in addition to mediating tissue fibrosis, TGF-*β* is widely implicated in the regulation of many biological responses, including cell proliferation, differentiation, apoptosis, autophagy, and immune response [25, 26]. Therefore, the direct approach of blocking TGF-*β*1 itself seems to bring a lot of potential adverse effects. [27]. By contrast, numerous studies have demonstrated that miRNAs are key players in normal kidney development, physiology, and pathogenesis of kidney disease and may therefore represent novel therapeutic to halt this most damaging process in CKD [16, 28, 29]. In vitro manipulation, activation of specific mRNAs in the kidney has been achieved by the delivery of inhibitors to block miRNA function or mimics to restore miRNA levels [30]. A number of studies in experimental animal models have obtained promising results in halting renal fibrosis by knocking down miR-21 [31], miR-214 [32], miR-433 [33], and miR-192 [34], or restoring expression of miR-29b [35, 36], and let-7b [18]. Furthermore, Wang et al. reveal that the genetic engineering of mesenchymal stem cells (MSCs) using miR-let7c enables effective antifibrotic effects and a high-efficiency delivery of therapeutic miR-let7c [37].

In this study, hsa-let-7c-5p expression level is markedly decreased in kidney biopsy specimens of CKD patients than that of the normal control group (*p* < 0.01), along with a marvelous increase in TGF-*β* (*p* < 0.001). Consistent with this finding, Brennan et al. reported that the downregulation of let-7c results in the induction of a variety of fibrotic effectors mediated by TGF-*β*1, including type I collagen *α*1 (COL1A1), type I collagen *α*2 (COL1A2), and thrombospondin (THBS1) [15]. Pezzolesi et al. reported that decreased circulating let-7c-5p was relevant to the increased risk of end-stage renal disease (ESRD) in type 1 diabetes patients [38]. However, we observed no significant differences in TGF-*β*R1 expression of CKD patients compared with the control group, which is inconsistent with prevalent opinion [12, 18]. It is likely that TGF-R1 is involved in other mechanisms that counteract this change. Correlation analysis shows hsa-let-7c-5p and TGF-*β* expression are negatively correlated with each other. This suggests that in the pathological process of renal fibrosis, downregulated hsa-let-7c-5p levels may play a biological function through its effect on the expression of TGF-*β* and relevant signaling. Several binding sites of hsa-let-7c-5p in the 3′ untranslated region (3′ UTR) of human TGF-*β* mRNA were identified through bioinformatics analyses. Dual-luciferase assay and ELISA were conducted to investigate the direct regulatory effect of hsa-let-7c-5p on TGF-*β* expression. It is confirmed that hsa-let-7c-5p can regulate the expression of TGF-*β* by directly binding to 167-173 position of its 3′UTR, thus affecting TGF-*β* signaling, and eventually influencing renal fibrosis and CKD development and progression.

Currently, the markers such as creatinine, eGFR, and proteinuria are routinely used to assess the development and progression of kidney injury [39]. Elevated serum creatinine level or decreased eGFR reflects an impaired kidney function. According to Table 1, we found that there were significant differences in the level of creatinine, cystatin C, urea nitrogen, hemoglobin, and albumin between CKD group and NC group. However, we observed no significant differences in the eGFR level of CKD group compared with the NC group, which may due to the fact that a majority of the CKD patients in this study were in the early stage of the disease. eGFR of these patients is often normal or mildly decreased [21].

Similarly, proteinuria is also associated with the development and outcomes of CKD. Proteinuria is regarded as the “golden standard” of early kidney injury. Hemmelgarn et al. found that at a given level of eGFR, the risks of death, myocardial infarction, and progression to kidney failure were independently increased in individuals with higher levels of proteinuria [40]. Hence, we analyzed serum creatinine, eGFR, proteinuria levels, and stage of CKD against hsa-let-7c-5p expression to further explore the potential relationship between the expression of hsa-let-7c-5p and clinicopathological factors in patients with CKD. We found that serum creatinine level was negatively related with the relative hsa-let-7c-5p expression level. Additionally, hsa-let-7c-5p expression level was found to be positively correlated with eGFR and the expression of hsa-let-7c-5p continuously decrease as proteinuria levels increase. Because of a relatively limited sample size, we divided CKD patients into two groups with a threshold of eGFR = 60 mL/min per 1.73 m^2^, a significant decrease in hsa-let-7c-5p levels of stage 3-4 CKD patients was observed, compared with that of early-stage patients. All these indicated that hsa-let-7c-5p level is related with CKD disease severity.

Growing evidence illustrates that miRNAs are both downstream effectors of TGF-*β*-dependent renal fibrosis and upstream regulators of TGF-*β*-dependent signaling [41, 42]. Feedback loops between TGF-*β* and miRNAs have heightened the focus on the use of miRNA-based therapies for the treatment of renal disease [30]. TGF-*β*1 induces transcription of several miRNA species that have antifibrotic effects including let-7c [12]. In this study, we demonstrate that hsa-let-7c-5p can directly regulate the expression of TGF-*β* by binding with its 3′UTR, thus affecting TGF-*β* signaling. Whether there is a feedback loop between TGF-*β* and hsa-let-7c-5p remains to be further investigated.

In conclusion, we identified that hsa-let-7c-5p has the potential to distinguish CKD patients from healthy controls and act as an indicator of disease severity. Meanwhile, our results elucidate the mechanism by which hsa-let-7c-5p regulates TGF-*β*1 expression, indicating that hsa-let-7c-5p modulation may be used as a therapeutic strategy for CKD. There are still several limitations in our research, such as the limited sample size and the lack of animal studies. Therefore, further studies are still needed to validate our findings before hsa-let-7c-5p is used as a biomarker for CKD early diagnosis in a clinical setting.

## 5. Conclusion

Changes in hsa-let-7c-5p expression and its target gene TGF-*β* are associated with the disease status of CKD. Let-7c-5p may contribute to the pathogenesis of renal fibrosis through TGF-*β* signaling, a potential diagnostic and therapeutic target of the disease.

## Figures and Tables

**Table 1:** Summary of demographics and clinical characteristics of donors.

Variables	Group		*p* value
	CKD	NC	
	(*N* = 23)	(*N* = 20)	
Gender			
Male, *n* (%)	16 (69.6%)	10 (50.0%)	
Female, *n* (%)	7 (30.4%)	10 (50.0%)	
Age (year)	42.9 ± 13.8	58.3 ± 12.2	0.001
Creatinine (*μ*mol/L)	105.9 ± 61.0	72.1 ± 11.1	0.021
eGFR (mL/min per 1.73 m^2^)	83.9 ± 33.9	95.4 ± 22.6	0.348
Cystatin C (mg/L)	1.4 ± 0.4	1.0 ± 0.2	0.005
Carbamide (mmol/L)	6.9 ± 5.0	4.7 ± 1.2	0.028
Urinary protein levels			
+, *n* (%)	4 (17.4%)		
++, *n* (%)	7 (30.4%)		
+++, *n* (%)	6 (26.1%)		
++++, *n* (%)	6 (26.1%)		
Hemoglobin (g/L)	125.6 ± 21.4	141.0 ± 15.8	0.01
Albumin (g/L)	29.9 ± 8.8	44.0 ± 3.0	<0.001
Classification of CKD			
G1, *n* (%)	11 (47.8%)		
G2, *n* (%)	4 (17.4%)		
G3a, *n* (%)	6 (26.1%)		
G3b, *n* (%)	1 (4.3%)		
G4, *n* (%)	1 (4.3%)		
G5, *n* (%)	0 (0%)		

Data are expressed as numbers with percentages in parentheses or as mean ± standard deviation. Classification of CKD, according to international guidelines. G1: eGFR≥90 mL/min per 1.73 m^2^, G2: 60 mL/min per 1.73 m^2^ ≤ eGFR≤89 mL/min per 1.73 m^2^, G3a: 45 mL/min per 1.73 m^2^ ≤ eGFR≤59 mL/min per 1.73 m^2^, G3b: 30 mL/min per 1.73 m^2^ ≤ eGFR≤44 mL/min per 1.73 m^2^, G4: 15 mL/min per 1.73 m^2^ ≤ eGFR≤29 mL/min per 1.73 m^2^, G5: eGFR<15 mL/min per 1.73 m^2^ [21].

**Table 2:** Relative expressions of hsa-let-7c-5p, TGF-*β*1, and TGF-*β*R1 determined by qPCR.

	NC (*n* = 20)	CKD (*n* = 23)	*p* value
hsa-let-7c-5p	1.00 ± 0.14	0.48 ± 0.08	0.0013
TGF-*β*1	1.00 ± 0.25	3.95 ± 0.66	0.0002
TGF-*β*R1	1.00 ± 0.14	1.26 ± 0.27	0.4117

Data are expressed as mean ± standard deviation.

## Data Availability

The data used to support the findings of this study are included in the article.
